# The Medullary Targets of Neurally Conveyed Sensory Information from the Rat Hepatic Portal and Superior Mesenteric Veins

**DOI:** 10.1523/ENEURO.0419-20.2021

**Published:** 2021-02-23

**Authors:** Cinthia Garcia-Luna, Graciela Sanchez-Watts, Myrtha Arnold, Guillaume de Lartigue, Nick DeWalt, Wolfgang Langhans, Alan G. Watts

**Affiliations:** 1 The Department of Biological Sciences, USC Dornsife College of Letters, Arts and Sciences, University of Southern California, Los Angeles, CA 90089; 2 Physiology and Behavior Laboratory, Eidgenössische Technische Hochschule Zürich, Schwerzenbach 8603, Switzerland; 3 Department of Pharmacodynamics, University of Florida, Gainesville, FL 32611; 4 Yale University, New Haven, CT 06520

**Keywords:** dorsal vagal complex, spinal cord, vagus nerve

## Abstract

Vagal and spinal sensory endings in the wall of the hepatic portal and superior mesenteric veins (PMV) provide the brain with chemosensory information important for energy balance and other functions. To determine their medullary neuronal targets, we injected the transsynaptic anterograde viral tracer HSV-1 H129-772 (H129) into the PMV wall or left nodose ganglion (LNG) of male rats, followed by immunohistochemistry (IHC) and high-resolution imaging. We also determined the chemical phenotype of H129-infected neurons, and potential vagal and spinal axon terminal appositions in the dorsal motor nucleus of the vagus (DMX) and the nucleus of the solitary tract (NTS). PMV wall injections generated H129-infected neurons in both nodose ganglia and in thoracic dorsal root ganglia (DRGs). In the medulla, cholinergic preganglionic parasympathetic neurons in the DMX were virtually the only targets of chemosensory information from the PMV wall. H129-infected terminal appositions were identified on H129-infected somata and dendrites in the DMX, and on H129-infected DMX dendrites that extend into the NTS. Sensory transmission via vagal and possibly spinal routes from the PMV wall therefore reaches DMX neurons via axo-somatic appositions in the DMX and axo-dendritic appositions in the NTS. However, the dearth of H129-infected NTS neurons indicates that sensory information from the PMV wall terminates on DMX neurons without engaging NTS neurons. These previously underappreciated direct sensory routes into the DMX enable a vago-vagal and possibly spino-vagal reflexes that can directly influence visceral function.

## Significance Statement

Vagal and spinal sensory nerves transmit chemosensory information from the hepatic portal and superior mesenteric veins (PMV) to the brain, eliciting central responses to control energy balance. The chemosensory signals are processed in the dorsal vagal complex (DVC), but the precise locations of PMV wall sensory terminals in the brain are unknown. Using the transsynaptic anterograde viral tracer HSV-1 H129, we identify a neuronal group that receive PMV wall chemosensory information: cholinergic neurons in the dorsal motor nucleus of the vagus (DMX) innervated by vagal and possibly spinal sensory inputs. These results provide the foundation for vago-vagal and spino-vagal chemosensory reflexes originating in the PMV wall.

## Introduction

The superior mesenteric vein (SMV), gastric vein (GV), and splenic vein (SV) form the venous system that provides the liver with nutrient-rich blood from the intestines, together with blood from the gallbladder, spleen, and parts of the pancreas and stomach. All of these veins drain into the hepatic portal vein (HPV), which then enters the liver. Various molecules, hormones, and ions have been infused into the HPV and their subsequent effects ascribed to spinal and vagal sensory nerve endings in the HPV wall, which then transmits this information to the brain. This mixture of sensory information includes: glucose (Niijima, 1969, 1989; Donovan et al., 1991a,b, 1994; Hamilton-Wessler et al., 1994; Niijima, 1996; Hevener et al., 1997), lipids (Randich et al., 2001; Cox et al., 2004), proteins (Mithieux et al., 2005; Duraffourd et al., 2012), amino acids (Niijima and Meguid, 1995), and ions (Morita et al., 1990; Bourque, 2008; Morita and Abe, 2011), leptin (Shiraishi et al., 1999), GLP-1 (Nakabayashi et al., 1996; Vahl et al., 2007), and CCK (Horn and Friedman, 2004). However, it is not entirely clear for all these substances that the HPV wall is the primary sensory site as opposed to the liver or a posthepatic site. Nonetheless, Saberi et al. (2008) have shown that HPV wall sensory endings extend into the SMV, thereby revealing that a key peripheral glucosensor is present in the walls of the hepatic portal and superior mesenteric veins (PMV) and transmits its information to the brain.

Vagal and spinal chemosensors in the PMV wall play key roles in several physiologic functions, including the control of glycemia and eating. In particular, they are implicated in initiating habitual meals in response to the premeal decline in glycemia (Campfield and Smith, 2003), and they are key components of the brain mechanisms that control sympathoadrenal responses to hypoglycemia in a rate-dependent manner (Donovan and Watts, 2014). Vagal sensory endings in the HPV wall originate primarily in the left nodose ganglion (LNG) and then run through its common hepatic branch (Berthoud, 2004). Spinal sensory endings derive from lower thoracic dorsal root ganglia (DRGs; T8–T13) and run via the splanchnic nerve through the celiac-superior mesenteric ganglion before reaching the PMV wall (Barja et al., 1983; Fujita and Donovan, 2005; Bohland et al., 2014).

Interoceptive signals are processed and integrated with other signals by neurons in the dorsal vagal complex (DVC), particularly the nucleus of the solitary tract (NTS) and the area postrema (AP; Rogers and Hermann, 1983; Menétrey and Basbaum, 1987; Rinaman et al., 1989; Rinaman and Schwartz, 2004). Although the NTS has been proposed as the key termination zone for sensory information from the HPV wall (Kobashi and Adachi, 1986; Morita et al., 1997), the detailed organization of the termination points of PMV wall sensory information in the brain remains unknown.

Conventional monosynaptic tracers are limited in their ability to identify hierarchically arranged synaptic connections within a network. Therefore, transsynaptic tracers are required to reveal the brain network associated with a specific target organ. The Herpes simplex virus-1 H129 is currently the only known transsynaptic viral tracer whose infection propagates predominantly in an anterograde direction (Archin and Atherton, 2002; Wojaczynski et al., 2015). It has therefore proved to be very effective for defining how sensory pathways from the stomach (Rinaman and Schwartz, 2004; Horn et al., 2014), adipose tissue (Song et al., 2009; Ryu et al., 2017), and trachea (McGovern et al., 2012, 2015) distribute within the brain.

We have taken advantage of these properties of H129 to determine the medullary targets of the chemosensory information that flows through the PMV vasculature. To do this we injected H129 into the wall of the HPV and SMV. The vagal sensory neurons that innervate the PMV originate in the LNG (Berthoud, 2004), and so we also injected H129 into the LNG of a separate group of animals. We did this to help identify how sensory nerves from the PMV wall relate to the population of vagal sensory neurons as a whole. These LNG injected animals were allowed to survive for between 1 and 3 d, thereby helping us to characterize H129 infection rates and patterns in the DVC. We then used double-immunohistochemistry (IHC) and triple-IHC followed by super-resolution confocal microscopy and 3D image analyses to identify the locations and chemical phenotype of infected neurons, the co-occurrence of H129 with neurochemical markers, and the nature and location of H129 terminal appositions in the DVC.

## Materials and Methods

### Animals

Sixty-seven adult male Sprague Dawley rats (Charles River) were used. At the time of surgery, they weighed between 211 and 330 g (mean 264 ± 3 g). They were housed two or three per cage in a climate-controlled environment (temperature 21 ± 1°C, 55 ± 5% relative humidity), on a 12/12 h light/dark cycle (zeitgeber time 0 = 11 P.M.) with unrestricted access to food (Standard laboratory chow, KLIBA 3433) and tap water for 10 d to allow adaptation to the housing conditions. Postsurgical animals were offered wet mash food in addition to chow. All animal procedures were approved by the Zurich Cantonal Veterinary Office.

### Surgical procedures

All animals were anesthetized with an isoflurane/oxygen mixture, and body temperature maintained between 37°C and 38°C throughout surgery with a heating pad. Atropine (0.05 mg/kg) was administered in the immediate presurgery period, and carprofen was administered postsurgery and on the following three days as an analgesic.

All animals were injected with herpes simplex virus-1 H129-772 (H129; The Center for Neuroanatomy with Neurotropic Viruses, Princeton University) engineered so that infected cells express enhanced green fluorescent protein (eGFP). All injections were made using a titer of between 8 and 9 × 10^8^ pfu/ml. The injectate contained 5% E133 (blue) food dye to ensure accurate placement into all targeted sites. All experimental and animal housing procedures involving H129 were performed using Biosafety Level 2 requirements.

Animals were initially divided into four groups: those with H129 injected into the wall of the PMV (*n* = 44), two control groups injected with H129 in the PMV lumen (*n* = 6), or onto the PMV surface (*n* = 5), and those injected with H129 into the LNG (*n* = 12). The PMV wall, the PMV lumen and the PMV surface groups were assigned either 96- or 120-h survival time; the LNG group was divided into 24-, 48-, or 72-h survival time (each *n* = 4). Survival times were based on those of Rinaman and Schwartz (2004), and on the difference in the distance needed for H129 to travel from the LNG or PMV into the medulla.

#### LNG H129 injections

**Figure 1.**
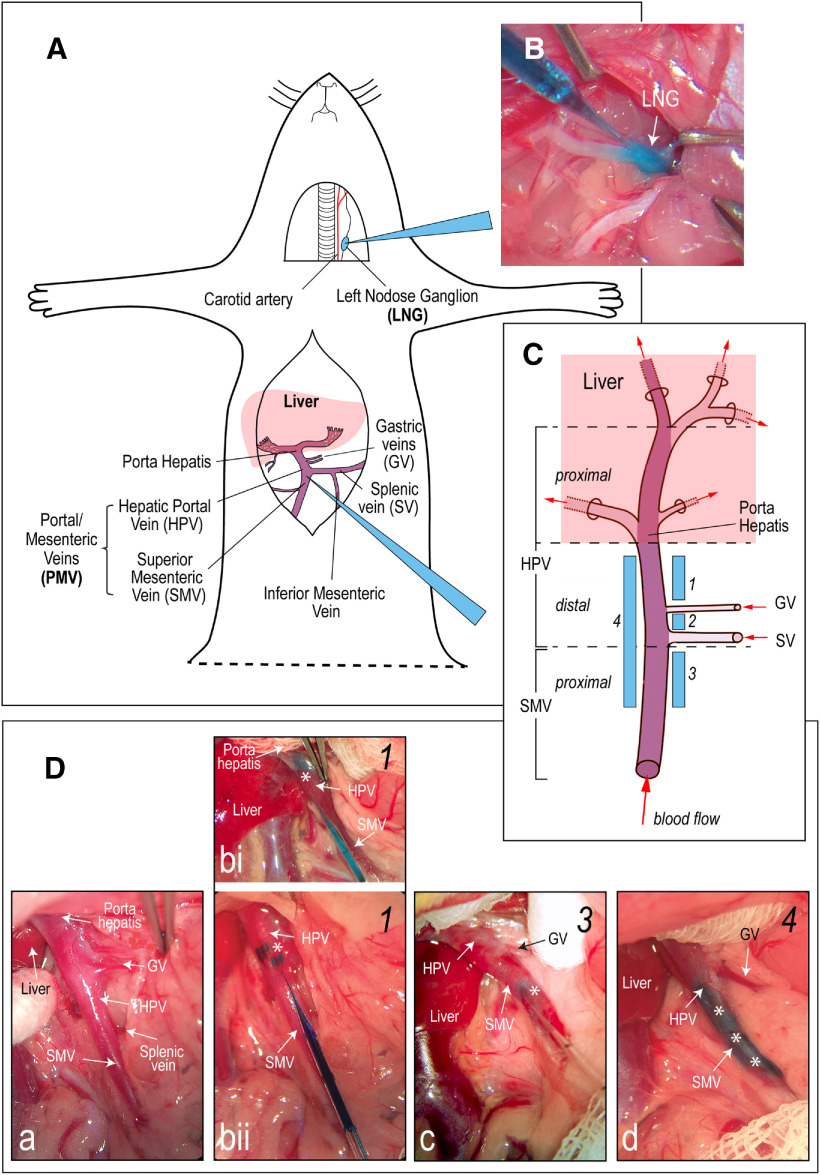
Experimental design. ***A***, Schematic representation of the two sites in male rats injected with the anterograde transsynaptic viral tracer, herpes simplex virus-1 H129 (H129): the LNG (*n* = 12), and the portal/mesenteric veins (PMV; *n* = 44) wall. ***B***, A photograph showing an H129 injection into the LNG. The micropipette injector is visible from the top left corner of the image with its tip in the LNG. The E133 blue dye allows the visualization of the injection. ***C***, Schematic representation of the HPV and its accompanying vasculature (adapted from Dong et al., 2010). The HPV is fed by the SMV that drains the small intestine, the GVs from the stomach, and the SV from the spleen, and parts of the pancreas and stomach. H129 injections were placed in the PMV wall at four locations (numbered 1–4) from the porta hepatis to the proximal SMV. ***Da***, A photograph showing the PMV wall regions injected with H129. Note that the SV, which marks the SMV/HPV transition, is only visible by retracting the overlying adipose tissue. It is only seen in this panel. Panels ***Db***–***Dd*** show three H129 injection sites (blue marks in the vein walls, highlighted by the white asterisks). Micropipette injectors are visible in panels ***b***, ***c***. Injection sites range from being close to the porta hepatis (***bi***) to the proximal SMV (***c***). A group 4 injection is shown in ***d***. The numbers in the top right corner of each panel correspond to the four PMV wall injection sites shown in ***C***. No image was available for group 2 injections.

Virus injections into the LNG was performed as previously described (Krieger et al., 2016, 2018; Lee et al., 2020). Briefly, a midline incision was made, and salivary glands, lymph nodes, sternohyoid, and omohyoid muscles were retracted to expose the trachea and carotid artery. The vagus nerve was separated from the carotid artery by blunt dissection until the LNG became accessible, and 0.9–1.2 μl of H129 was injected using a glass capillary micropipette attached to a microinjector (Picospritzer III injector; Parker Hannifin; Fig. 1*A*,*B*). Results from the LNG-injections described here are from a group of animals that were also used in a previous study (Krieger et al., 2018).

#### PMV wall H129 injections

The PMV caudal to the porta hepatis was exposed and made accessible by way of a 5-cm skin incision in the rostral-caudal midline using a procedure similar to one for implanting HPV catheters (Arnold et al., 2012). The skin was separated by blunt dissection from the underlying muscle, and a 5 cm laparotomy was made beneath the skin incision. The small and large intestines were exteriorized, placed on the animals left side, and covered with a warm-saline-moistened gauze. The liver was retracted toward the diaphragm and secured with a piece of wet gauze.

The target sites in the PMV wall were carefully manipulated and elevated with fine forceps. A glass capillary micropipette containing H129 was introduced into the PMV wall parallel to the lumen and directed toward that porta hepatis. H129 was then injected into various sites of the PMV wall using a microinjector (Picospritzer III injector; Parker Hannifin). Four sets of injections were made to target: (1) the HPV wall between the porta hepatis and GV, *n* = 13; (2) the HPV wall between the GV and SV, *n* = 8; (3) the proximal SMV wall, *n* = 8; and (4) the PMV wall between the porta hepatis and the proximal SMV, *n* = 15 (Table 1; Fig. 1*C*,*D*). The various sets of observations from H129 injections in the LNG and PMV wall that we report in the Results were each obtained from at least three animals.

**Table 1:** Injection targets in the HPV and SMV walls

Group	Target	Number injected	Injection volume (μl)	Positive brain infections (%)
	PMV wall			
1	HPV wall: from the porta hepatis to the GVs	13	1.0–1.4	46
2	HPV wall: between the GV and SV	8	2.0	50
3	SMV wall: upstream from the SV	8	0.8–1.8	63
4	HPV + SMV wall: from the porta hepatis to the proximal SV	15	1.5–4.5	40
	PMV lumen	6	2.0	0
	PMV surface	5	2.0	20^*^

Four regions of the HPV and SMV walls were injected with H129. See Figure 1 for the details of these locations. In addition, two control regions, the HPV lumen and surface were administered with H129.

* One PMV surface animal showed positive H129 in the same dorsal medulla regions as the PMV wall injections. However, the injector tip of this animal punctured the surface of the PMV wall, making it likely that H129 infected the same sensory endings as those animals in the PMV wall groups.

After the designated survival times, rats were anesthetized with isoflurane (IsoFlo; Abbott, Baar, Switzerland) and transcardially perfused with 0.9% saline followed by pH 9.5 borate-buffered-4% paraformaldehyde. Brains of all animals, both nodose ganglia and DRGs (T8–T13) of PMV wall animals were excised. Brains were postfixed in borate-buffered-4% paraformaldehyde + 15% sugar for 24 h at 4°C, fast frozen in dry ice and stored at −70°C until further processing. Both nodose ganglia, and DRGs were postfixed for 2 h and cryoprotected in borate-buffered-4% paraformaldehyde + 25% sugar solution for 2 d, before embedding in OCT, frozen with dry-ice and stored at −70°C until further processing.

Four series of frozen brain coronal sections (30 μm) were cut throughout the rostrocaudal extent of the DVC, using a sliding microtome, and stored in a cryoprotectant solution (50% 0.05 m sodium-phosphate buffer, 30% ethylene glycol, 20% glycerol) until IHC was performed. Both nodose ganglia, and DRGs were cut at −20°C with a CM1850 cryostat (Leica Microsystems Inc.) in 10-μm sections (1:4), thaw-mounted onto Superfrost Plus slides and kept at −70°C until IHC was performed.

### Immunohistochemistry

#### Antibody characterization

##### Chicken anti-GFP (eGFP)

Ab13970 (Abcam) was used. Manufacturer determined the specificity through the use of immunofluorescence histochemistry, where eGFP-immunoreactivity (-ir) was absent in non-transfected mouse embryo fibroblast NIH/3T3 cells compared with that of eGFP-transfected NIH/3T3 cells. Additionally, no eGFP-ir was detected in wild-type BDNF mice cortical cultures in contrast to those transfected with BDNF-eGFP (Wosnitzka et al., 2020).

##### Mouse anti-dopamine-β-hydroxylase (DBH)

MAB308 (Millipore) was used. The manufacturers reported that western blot analysis recognized a band of ∼70 kDa in rat tissue (Bruinstroop et al., 2012). In addition, immunolabeling was abolished following preabsorption with the immunogen (Rinaman, 2001).

##### Goat anti-choline acetyltransferase (ChAT)

AB144P (Millipore) was used. This antibody was raised against the human placental enzyme. The manufacturers reported that western blots of mouse brain lysates showed a band at ∼70–74 kDa. In the current study, the pattern of labeling seen in the DVC is in accordance to previously described cholinergic cell groups (Ruggiero et al., 1990).

##### Rabbit anti-synapsin I pSer 62/67 (SynI)

AP08742PU-N (Acris Antibodies Inc.) was used. The manufacturers reported that western blot of rat cortical lysates showed immunolabeling of a band at 78 kDa, a signal that is blocked by preabsorption with the phospho-peptide used as antigen (manufacturer’s information).

##### Biotinylated *Griffonia simplicifolia* Lectin I isolectin B4 (IB4)

B-1205 (Vector Laboratories) was used. This Isolectin has an appropriate number of biotins bound to provide optimum staining characteristics for this lectin (as per manufacturer’s description). This conjugate is supplied essentially free of unconjugated biotins and it is used here as a marker of unmyelinated vagal sensory nerves.

#### Secondary antibodies

Donkey anti-chicken IgG-Alexa Fluor 488 (catalog #703-545-155), donkey anti-mouse IgG-Cyanine 3 (catalog #715-165-150), donkey anti-goat IgG-cyanine 3 (catalog #705-165-147), donkey anti-goat IgG-Alexa Fluor 647 (catalog #705-605-147), donkey anti-rabbit IgG-Alexa Fluor 647 (catalog #711-605-152), and streptavidin-cyanine 3 (catalog #434315; Thermo Fisher Scientific, Invitrogen) were used. Unless stated, all secondary antibodies were obtained from Jackson ImmunoResearch. The specificity of all secondary antibodies was assessed by incubating control sections without primary antibodies followed by incubation with the corresponding secondary antibody. None of these control incubations resulted in immunofluorescent labeling.

##### IHC on brain sections

Brain sections were taken out of cryoprotectant, washed five times for 5 min each at room temperature in Tris-buffered saline (TBS; pH 7.4), and processed for double or triple fluorescent IHC.

##### Double-IHC: eGFP + IB4

After rinsing in TBS, sections were rinsed in PBS for 10 min, incubated in a PBS-blocking solution containing 2% (v/v) normal donkey serum (NDS; Chemicon), 0.2% (v/v), Triton X-100 (Sigma-Aldrich), and 1 mg/ml bovine serum albumin (Sigma-Aldrich) for 45 min at 30°C. They were then transferred to a cocktail of chicken anti-eGFP (1:5000) and biotinylated IB4 (1:100) diluted in the PBS-blocking solution, incubated for 1 h at 30°C, and then transferred to 4°C for an overnight incubation. Sections were washed in PBS five times for 5 min each, and incubated at room temperature for 1 h in a cocktail of donkey anti-chicken IgG-Alexa Fluor 488 (1:500) and streptavidin-cyanine 3 (1:1000) in the PBS-blocking solution. Sections were again washed in PBS, mounted onto Superfrost Plus slides and allowed to air dry in the dark. Once dry, all slides were coverslipped with an antifade solution of 50% glycerol/50% PBS/2% DABCO (1,4-Diazabicyclo[2.2.2]octane), and stored in the dark at 4°C.

##### Triple-IHC: eGFP + DBH + ChAT

After rinsing in TBS, sections were incubated in a blocking solution of TBS/2% NDS/0.1% Triton X-100 for 3 h at room temperature. Sections were transferred to the primary antibody cocktail of chicken anti-eGFP (1:10,000), mouse anti-DBH (1:10,000), goat anti-ChAT (1:1000) diluted in the TBS-blocking solution, and incubated for ∼68 h at 4°C. Sections were removed from the primary antibody mix, washed in TBS, and transferred to a secondary antibody cocktail of donkey anti-chicken IgG-Alexa Fluor 488, donkey anti-mouse IgG-cyanine 3 and donkey anti-goat IgG-Alexa Fluor 647 (all 1:2000) in the TBS-blocking solution, and then incubated overnight at 4°C. Sections were rinsed in TBS five times for 5 min each, mounted, coverslipped, and stored as described above.

##### Triple-IHC: eGFP + Synl + IB4

This was accomplished in two consecutive stages: sections were first processed to completion for eGFP-ir and SynI, and then processed for IB4. For the first incubation, sections rinsed and blocked as above in the TBS-blocking solution for 3 h at room temperature, transferred to a primary antibody cocktail of chicken anti-eGFP (1:10,000) and rabbit anti-SynI (1:10,000) diluted in the TBS-blocking solution and incubated for ∼68 h at 4°C. Sections were removed from the primary antibody mix, washed in TBS, and transferred to a secondary antibody cocktail of donkey anti-chicken IgG-Alexa Fluor 488 and donkey anti-rabbit IgG-Alexa Fluor 647 (both 1:2000) in the TBS-blocking solution, and incubated overnight at 4°C. From here on, sections were processed in the dark to preserve the fluorescent labeling. They were rinsed in TBS five times for 5 min each, rinsed in PBS for 10 min, then incubated in a blocking solution of the PBS-blocking solution for 45 min at 30°C, followed by a 1-h incubation at 30°C in a solution of biotinylated IB4 diluted 1:100 in the PBS-blocking solution before being transferred for an overnight incubation at 4°C. Sections were washed in PBS five times for 5 min each, then incubated at room temperature for 1 h in a solution of streptavidin-cyanine 3 (1:1000) in the PBS-blocking solution. Sections were washed in PBS, mounted, coverslipped, and stored as described above.

##### Nodose and DRGs IHC: eGFP + ChAT

Slides were warmed up to room temperature, placed on a hotplate for 5 min at 35°C, rehydrated in TBS for 5 min, then incubated in the TBS-blocking solution for 2 h at room temperature. Slides were transferred to a primary antibody cocktail of chicken anti-eGFP (1:5000) and goat anti-ChAT (1:500) diluted in the TBS-blocking solution, incubated for ∼24 h at 4°C. Slides were removed from the primary antibody mix, washed in TBS, and transferred to a secondary antibody cocktail of donkey anti-chicken IgG-Alexa Fluor 488 (1:500), and donkey anti-goat IgG-cyanine 3 (1:500) in the TBS-blocking solution, and incubated overnight at 4°C. Slides were then rinsed in TBS five times for 5 min each, allowed to dry in the dark, coverslipped using 50% glycerol/50% PBS/2% DABCO, and stored in the dark at 4°C

### Image acquisition

#### Epifluorescence imaging

Each of the selected fields within a section was imaged by acquiring tiled low-power scans that captured each fluorescent channel and the corresponding darkfield illumination resulting in up to four images per section. Image acquisition used a motorized Zeiss AxioImager Z1 microscope (Carl Zeiss MicroImaging Inc.) equipped with a Hammamatsu Orca ER camera using Fluar 5× (numerical aperture 0.25) and Plan Apochromat 10× (numerical aperture 0.45) objectives. Both the image capture and tiling processes (using a 10% overlap between neighboring tiles) was controlled by Volocity software (version 6.1; PerkinElmer). Adobe Photoshop 2020 (Adobe Systems Inc.) was used postcapture to create composite images, including whole image contrast and/or brightness modifications where appropriate.

#### Confocal imaging

High-power confocal Z-stack tile images were captured using a LSM700 Confocal Laser Scanning Microscope controlled by Zeiss Zen software using Plan Apochromat 20× (numerical aperture 0.8) and EC Plan-Neofluar 40× oil-corrected (numerical aperture 1.3) objectives. Images were taken using a unidirectional line scan at 1024 × 1024 pixels captured on an eight-bit gray scale. Each pixel was scanned twice and the result was averaged to reduce optical noise. Appropriate emission filters were applied to prevent bleed-through between optical channels. Images were stitched with Zen software using a 10% overlap between frames. 2D flatten confocal Z-stack images were constructed by maximum intensity projection and by taking images through the *z*-axis with a frequency of 5 μm throughout the entire depth of the tissue section (30 μm). Images were contrast-enhanced using Adobe Photoshop 2020, and then used to create a composite image including two or three fluorescent channels.

##### Super-resolution confocal microscopy

Super-resolution images were captured using a Leica SP8 with DIVE Confocal Laser Scanning Microscope controlled by LAS/X software (Leica Microsystems Inc.) using an HC PL APO 63× oil-corrected (numerical aperture 1.4) objective. A digital zoom of 2.23 was applied using the LAS/X software to result in an image magnified to 140.5×. Images were taken using a bidirectional line scan at 512 × 512 pixels captured on a 16-bit gray scale, each pixel was scanned twice and the result was averaged to reduce optical noise. White light laser tuned to 488-, 561-, and 647-nm excitation and 497- to 519-, 578- to 591-, and 692- to 761-nm collection bandwidths were used to image sequentially. The collection was at 2.5× Nyquist oversampling for Lightning deconvolution from Leica. 3D images were constructed by taking images of planes through the *z*-axis with a frequency of 0.13 μm through a 13-μm depth or 0.23 μm throughout the entire 30-μm depth of the tissue section, using LAS/X software. Contrast enhancement was performed to images using Adobe Photoshop 2020. We analyzed the co-occurrence (Dunn et al., 2011; Johnson et al., 2018) of eGFP, IB4, and SynI in fluorescently-labeled structures in a selected region of interest in the commissural NTS and in the dorsal motor nucleus of the vagus (DMX). We first set thresholds to define specific and background signals, and then we quantified the total number of SynI structures and their mean diameter was calculated using the 3D analysis module of LAS/X software. Subsequently, a threshold of one standard deviation below and above the mean diameter (0.27–0.98 μm) was used to build a protocol that identified double-labeled and triple-labeled structures that contained SynI.

### Atlas-based mapping

Tissue sections corresponding to atlas levels (AL) 63–73 were analyzed, (Swanson, 2018). The left hemisphere of animals’ brain is on the left side of the images. Dark-field photomicrographs of the same sections used to image eGFP, DBH and ChAT immunostaining were produced with a Zeiss AxioImager Z1 microscope using a Hammamatsu Orca ER camera and controlled by Volocity software. Brightness and contrast adjustments were made with Adobe Photoshop 2020 before mapping, and all photomicrographs, including eGFP, DBH and ChAT immunostaining, and dark-field images, were imported to Adobe Illustrator 2020 (AI). Tracts and anatomical regions were identified and outlined with an overlying layer of the AI file of the corresponding AL, using the dark-field photomicrography along with DBH and ChAT immunostaining, to help delineate catecholaminergic and cholinergic medullary regions. To map H129-infected neurons and fibers, circles and lines were drawn in an AI layer over every eGFP-labeled structure. After the mapping, a merged maximum projection intensity image of the same section used to map, that included eGFP, DBH and ChAT immunostaining, was used to identify eGFP single-labeled neurons, eGFP/DBH or eGFP/ChAT double-labeled, and eGFP/DBH/ChAT triple-labeled neurons. Cells and fibers representations were then superimposed onto the corresponding AL template, creating atlas-based maps of the DVC of PMV wall H129-injected rats.

### Statistics

Body weight (BW) data are expressed as mean ± SEM. Changes in BW between groups along survival times are expressed as percentage change in BW from the day of surgery. Statistically significant changes (*p* < 0.05) were detected by one-way ANOVA (JMP Pro 14 Software, SAS Institute Inc.).

## Results

### The effects of H129 infections on BW

All H129-infected animals remained asymptomatic at all survival times as assessed by coat condition, locomotion, and signs of general malaise. PMV wall-injected rats increased their mean BWs by 8.3% 120 h after surgery compared with their presurgery BWs (Table 2; *F*_(4,189)_ = 30.48, *p* = 1.40178E^−19^). Although PMV lumen and surface administered animals showed increased BWs during their survival period, these were not significant from their surgery day weights most likely because of the variance within the small group number. In contrast, the mean BWs of LNG-injected rats decreased by 8% compared with presurgery BW after 72 h (Table 2; *F*_(3,32)_ = 6.22, *p* = 0.0018).

**Table 2.** Percentage change in BW from surgery

Target	Preinjection BW (g)	Survival times				
		24 h	48 h	72 h	96 h	120 h
PMV wall	263.9 ± 3.4	0.8 ± 0.4	2.9 ± 0.5*	4.6 ± 0.5*	6.3 ± 0.5*	8.3 ± 0.6*
PMV lumen	268.0 ± 6.9	−0.5 ± 1.0	0.9 ± 0.9	2.7 ± 0.8	5.8 ± 0.5	8.5 ± 1.2
PMV surface	265.3 ± 8.8	0.5 ± 2.0	2.6 ± 1.2	4.5 ± 1.4	7.8 ± 1.3	9.1 ± 1.6
LNG	297.7 ± 5.4	−0.9 ± 0.7	−4.9 ± 1.3*	−8.0 ± 3.3*		

Percentage of BW changes along survival times compared with the preinjection BW. Data of preinjection BW are expressed as the mean ± SEM in grams (PMV wall *n* = 44; PMV lumen *n* = 6; PMV surface *n* = 5; LNG *n* = 12). Values of change of BW along the survival times are expressed as the mean ± SEM in percentages. One-way ANOVA showed differences between survival times, PMV wall group *p = 1.40178E^−19^ and LNG group *p = 0.0018 versus their own preinjection BW.

### H129 brain infections, general observations

The presence and extent of H129-infected cells in the DVC after PMV wall injections varied greatly between individual cases. These ranged from animals that had no discernable infections, animals with only one or two neurons, to those that had substantial infections. Nonetheless, the distribution of infected glial cells, neuronal soma, dendrites, and axons was virtually identical between all animals in the same experimental group. However, three PMV wall-injected animals did have H129 infection patterns that were completely different from all others, and so were excluded from further analyses. One of the five PMV surface-injected animals had infection in the brain, the pattern of which was identical to the PMV wall-injected animals. We attribute this infection to H129 infiltrating into the PMV wall, which in this animal was damaged by the microinjector during the surface application. No PMV lumen-injected animals had infections in the brain. Table 1 shows that the infection efficiency of PMV wall injections appeared to vary according to the targeted site, with those in the SMV wall (group 3) being more effective (63%) than those proximal to the porta hepatis (group 1; 46%).

**Figure 2.**
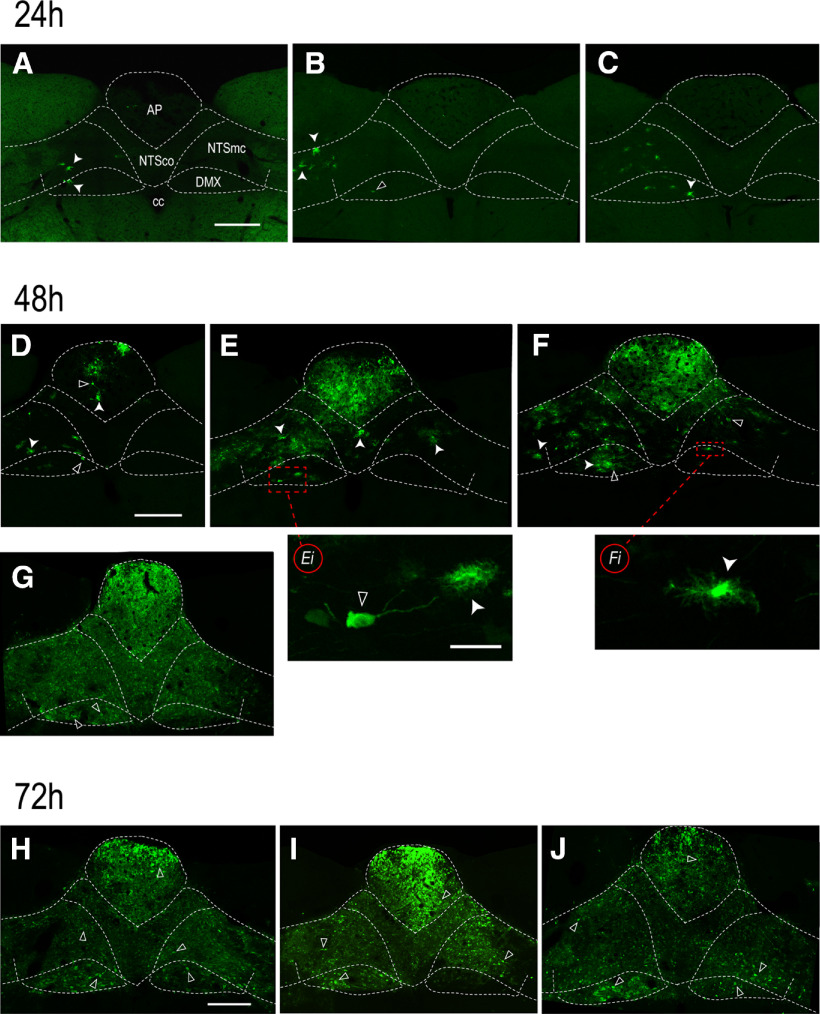
The distribution of H129 infections in the DVC after 24, 48, or 72h survival times following H129 injections in the LNG. The dorsal medulla of rats 24h (***A–C***), 48h (***D–G***), or 72h (***H–J***) after H129 injections into the LNG. Each panel (***A–J***) corresponds to a different animal. Closed arrowheads show eGFP-ir in H129-infected astrocytes; open arrowheads show eGFP-ir in H129-infected neurons. 24h, Astrocytes are the first cells to be infected after H129 arrives into brain in LNG sensory neurons. A few cells are evident in the AP (***A***, ***B***), NTS, and DMX (***A–C***) 24h after LNG injections. One animal in this group had no apparent infection of any kind (data not shown). 48h, Infected neurons are apparent together with infected astrocytes 48h after H129 injections. These cells are evident in the NTS and DMX of all four animals (***D–G***; and at higher magnification in ***Ei***, ***Fi***). Their numbers are highly variable between individuals despite the same survival times. Panel ***E*** shows the adjacent section from the 48-h animal in Figure 3. 72h, By 72h, large numbers of infected neurons are seen in the AP, NTS, and DMX, particularly the left DMX. Scale bars: 100 μm (***A–J***) and 50 μm (***Ei***, ***Fi***). AP, area postrema; cc, central canal; DMX, dorsal motor nucleus of the vagus nerve; NTSco, commissural part of the nucleus of the solitary tract; NTSmc, caudal subzone of the medial part of the NTS.

Although the PMV wall-injected animals had 96- and 120-h survival times, the large variation in infection intensities between animals precluded our ability to assess any time-dependent infection progression pattern. However, our LNG injections did provide some appreciation of how H129 infections progressed through connected brain regions. Ten from a total of twelve LNG-injected animals had eGFP-ir in the DVC. One animal from each of the 24- and 72-h groups had no detectable eGFP-ir in the DVC. Figure 2 shows that the earliest evidence of infection was increased in eGFP-ir in what appeared to be astrocytes in the NTS, AP, and DMX after 24-h survival (Fig. 2*A–C*). As the infection progressed to 48 h (Fig. 2*D–G*), the numbers of eGFP-ir neurons increased in these same regions, but most notably in the AP. Astrocytes with eGFP-ir were still seen in some regions at this time (Fig. 2*Ei*,*Fi*).

By 72 h (Fig. 2*H–J*), substantial numbers of eGFP-ir neurons were now evident in the NTS, AP, and DMX, although no 72-h animal appeared to be more heavily infected than the 48-h animal shown in Figure 2*G*. These results show that the time course for H129 infections varies from animal to animal even when the same target is injected. For example, the extent of infections in 48-h survival animals varied from scant (Fig. 2*D*) to substantial (Fig. 2*G*). These two animals were injected on the same day with the same volume and virus titer, and were perfused after the same survival time. This means that the correlation between the infection extent and survival time is not tight (also see Lo and Anderson, 2011).

### Comparing the distribution of H129-infected neurons after injections in the PMV wall or the LNG

**Figure 3.**
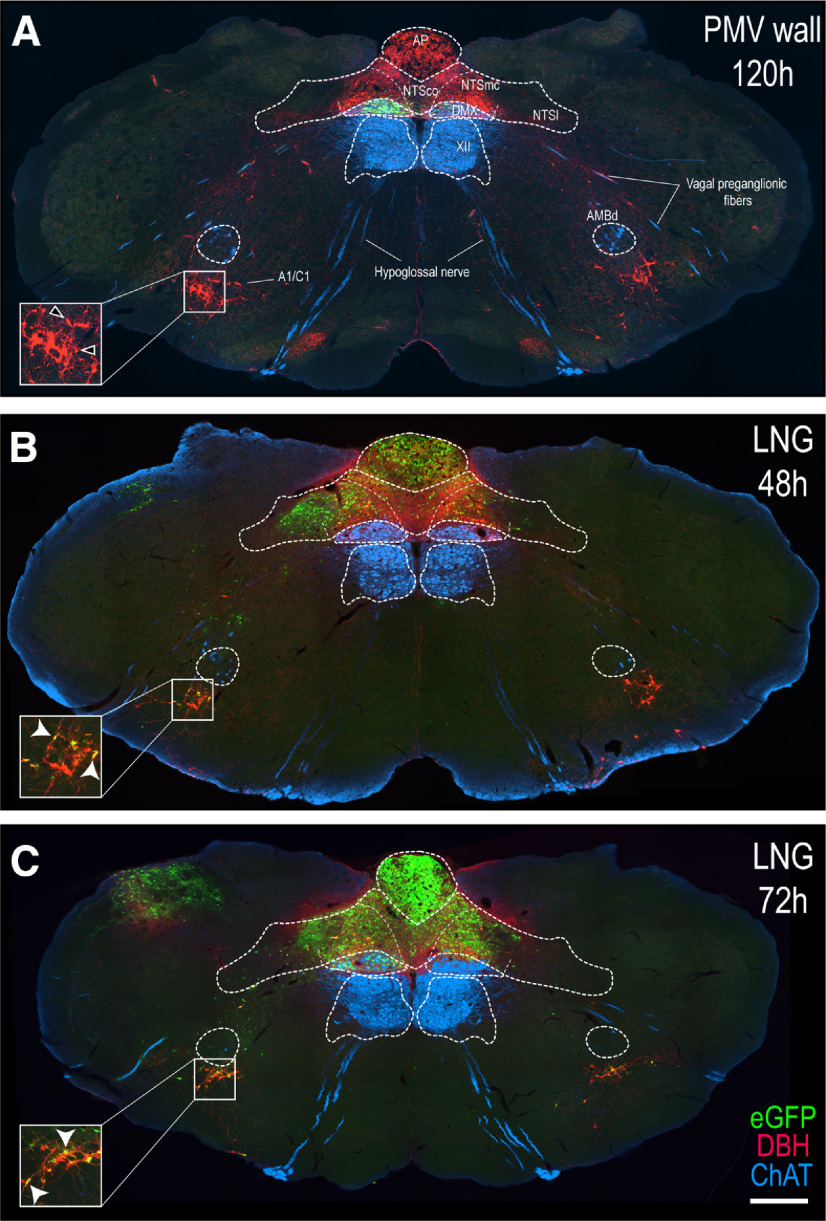
Differential distribution of H129 infection in the hindbrain of PMV wall-injected and LNG-injected rats. Epifluorescence photomicrographs of PMV wall-injected animals after 120-h survival (***A***), and LNG-injected rats with a survival times of 48 h (***B***) or 72 h (***C***), indicating H129 infection (eGFP, green channel), catecholaminergic (DBH, red channel), and cholinergic (ChAT, blue channel) neurons. Note that in PMV wall injection (***A***), eGFP-ir labeling is restricted to the DMX, whereas in LNG injections, H129 infection starts at the AP and NTS (***B***), and it progresses until it reaches also the DMX (***C***). Insets show higher magnification images of A1/C1 cell group indicated by the white boxes. Single DBH-labeled neurons are shown with the open arrowheads, and double-labeled eGFP + DBH neurons are shown with white arrowheads. Scale bar: 200 μm. A1/C1, noradrenergic/adrenergic cell group A1; AMBd, dorsal division of the ambiguus nucleus; AP, area postrema; DMX, dorsal motor nucleus of the vagus nerve; NTS, nucleus of the solitary tract; NTSco, commissural part of the NTS; NTSl, lateral part of the NTS; NTSmc, caudal subzone of the medial part of the NTS; XII, hypoglossal nucleus.

**Figure 4.**
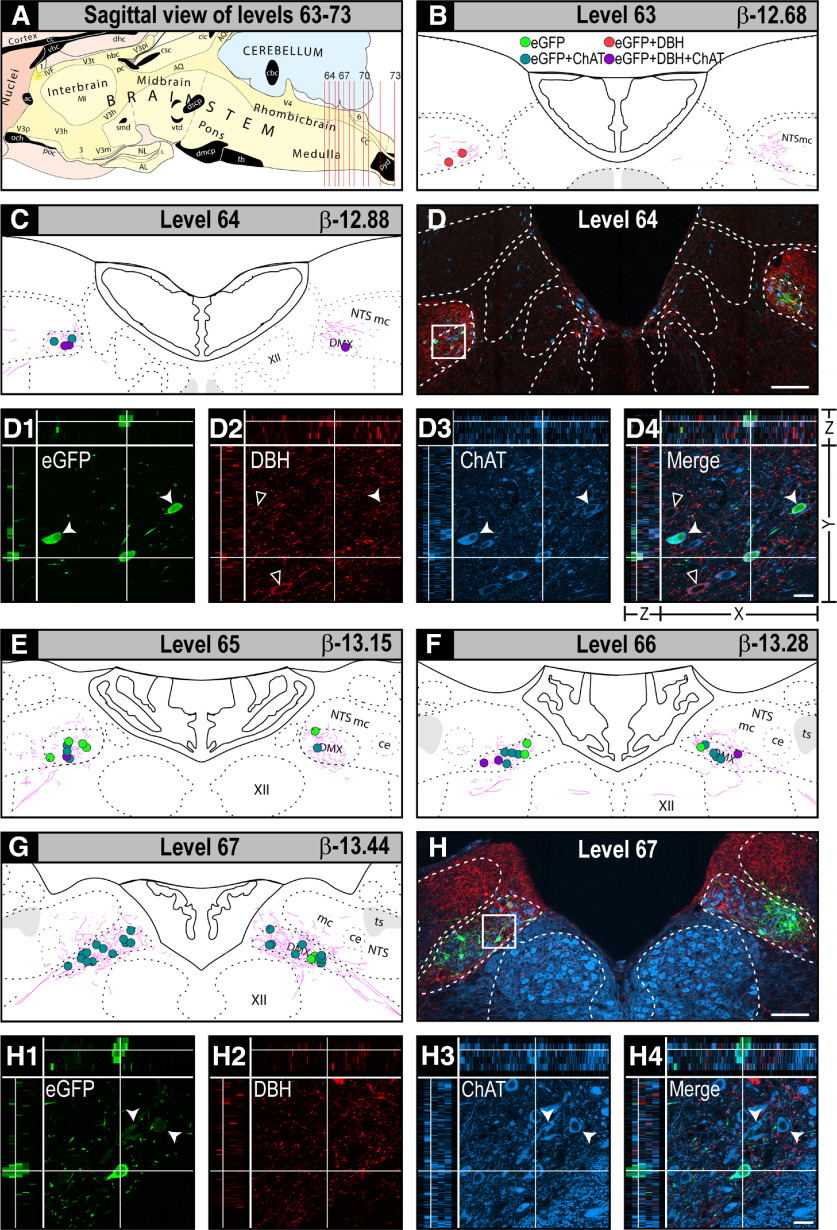

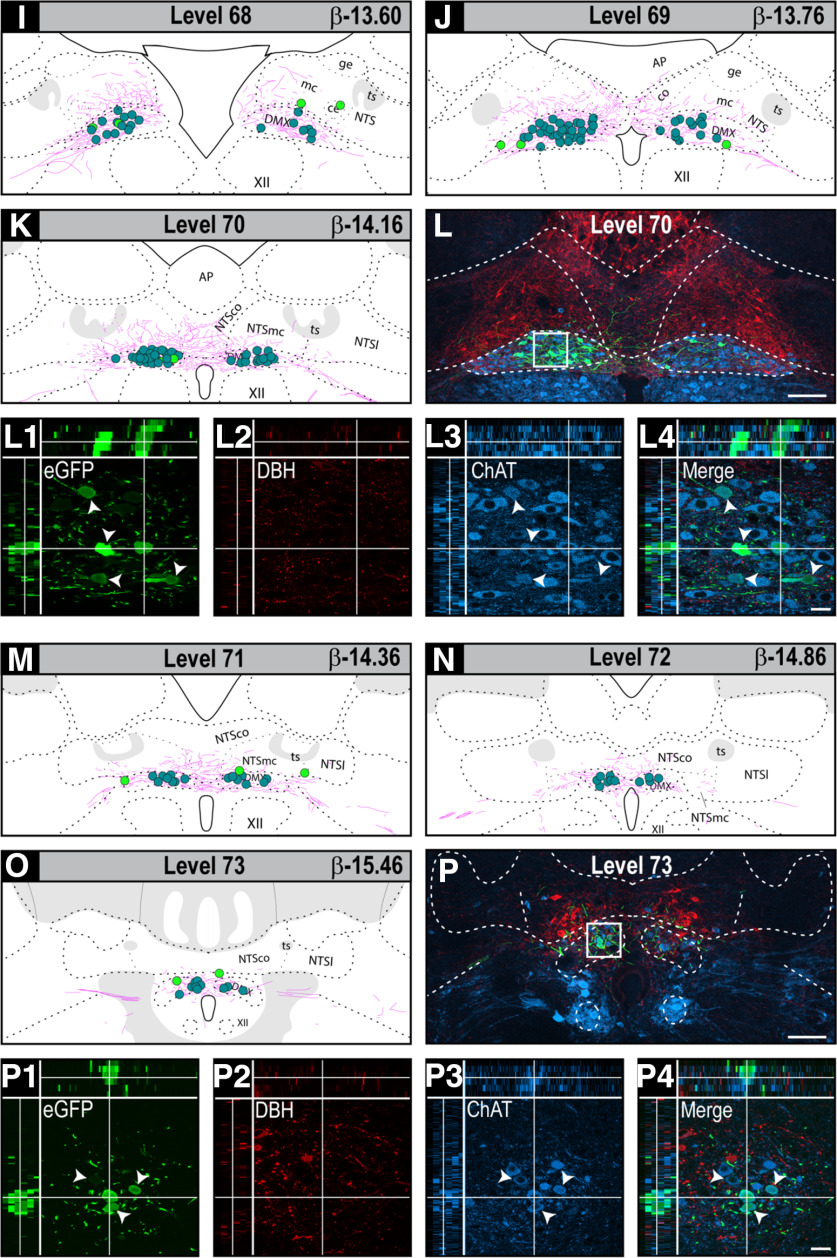
The locations of H129-infected neurons and their co-occurrence with catecholaminergic and cholinergic neurons in the DVC of PMV wall-injected rats. Locations eGFP-ir neurons in the DVC after H129 injections in the walls of the PMV. Locations and co-occurrence results are presented in two ways. First plotted onto maps of levels 63–73 from the Swanson Rat Brain Atlas (Swanson, 2018). Panel ***A*** shows a sagittal view of the caudal brainstem indicating rat brain AL 63–73 that cover the entire dorsal DVC. Individual levels are shown in the following panels: (***B***) 63, (***C***) 64, (***E***) 65, (***F***) 66, (***G***) 67, (***I***) 68, (***J***) 69, (***K***) 70, (***M***) 71, (***N***) 72, and (***O***) 73. The maps also show locations of neurons where eGFP-ir co-occurred in neurons containing DBH-ir and/or ChAT-ir. Maps are plotted as composites to represent the locations of H129-infected neurons at each AL. They derive from all infected animals. The map color codes are shown in ***B*** and are as follows: green dots, eGFP-ir somata; red dots, double-labeled eGFP-ir + DBH somata; cyan dots, double-labeled eGFP + ChAT somata; purple dots, triple-labeled eGFP + DBH + ChAT somata; pink lines, eGFP-ir neuronal processes. Each dot indicates one neuron. Second, confocal photomicrographs of sections of locations from four selected rat brain AL (***D***) 64, (***H***) 67, (***L***) 70, and (***P***) 73. These same panels also show higher magnification confocal maximum projections of selected DVC regions to illustrate co-occurrence. The white squares show the sampled region observed in panels ***D***, ***H***, ***L***, and ***P***. They are labeled 1–4 in each case. Green channel, eGFP; red channel, DBH; blue channel, ChAT. Panels 1–4 in ***D***, ***H***, ***L***, ***P*** also show the three-plane orthogonal view obtained from their confocal Z-stacks. Each panel 1 shows structures labeled for eGFP (***D1***, ***H1***, ***L1***, ***P1***); panel 2 shows structures labeled for DBH (***D2***, ***H2***, ***L2***, ***P2***); panel 3 shows structures labeled for ChAT (***D3***, ***H3***, ***L3***, ***P3***); and panel 4 the respective merged images, where single labeled structures are green, red, or blue and co-occurring structures in cyan, yellow, or purple (***D4***, ***H4***, ***L4***, ***P4***). Double-labeled or triple-labeled neurons that contain eGFP are shown with the white arrowheads, and double-labeled DBH + ChAT neurons are shown with open arrowheads. The arrangement of the *x*-, *y*-, and *z*-axes is shown on panel ***D4***, and the horizontal and vertical white lines in panels 1–4 of ***D***, ***H***, ***L***, ***P*** target a representative double or triple-labeled structures in the *x*-, *y*-, and *z*-axes. Scale bars: 100 μm (***D***, ***H***, ***L***, ***P***) and 20 μm (panels 1–4 of ***D***, ***H***, ***L***, ***P***). AP, area postrema; DMX, dorsal motor nucleus of the vagus nerve; NTS, nucleus of the solitary tract; NTSce, central part of the NTS; NTSco, commissural part of the NTS; NTSge, gelatinous part of the NTS; NTSl, lateral part of the NTS; NTSmc, caudal subzone of the medial part of the NTS; ts, solitary tract; XII, hypoglossal nucleus.

Figure 3*A* shows that in PMV wall-injected animals the distribution of H129 infection after 120 h was restricted to the DMX with only very occasional infections seen in the NTS most of which were adjacent to its border with the DMX (see also Fig. 4).

Most identifiable H129-infected cells in LNG-injected rats with the shortest survival time (24 h) were glial cells in the NTS, although one neuron was evident in the DMX (Fig. 2*A–C*). As expected with the two longer survival times (48 and 72 h), LNG-injected animals had significantly more extensive neuronal infections in the DVC than in any PMV wall-injected animals.

Figure 3*B* shows that after 48-h survival, LNG-injected animals display a distribution of H129-infected neurons in the DVC that is characteristic of vagal sensory inputs, the vast majority of which were found in the left lateral, commissural, and caudal NTS. The AP was also heavily infected. Furthermore, the A1/C1 catecholaminergic cell group in the left ventrolateral medulla (VLM) showed H129-infected neurons that co-occurred with DBH (Fig. 3*B*). At this DVC level (AL 69–70) in this particular animal no H129-infected neurons were evident in the DMX (Fig. 3*B*). However, Figure 2*D–G* shows that H129-infected neurons were present at this and other levels of the DMX of all 48-h animals, including the animal shown in Figure 3*B* (Fig. 2*E*).

After 72-h survival, Figure 3*C* shows that in addition to the infected regions seen in the 48-h LNG rats, H129 infection progressed to the right NTS and VLM, and to the left DMX, and was much heavier than in most animals surviving for 48 h. We note that the extent of H129 infections did not always correspond to survival times. Thus, one 48-h animal (Fig. 2*G*) showed at least as extensive a degree of H129 infection as the 72-h animals (Fig. 2*H–J*).

### Distribution H129 in the DVC of PMV wall-injected rats

**Figure 5.**
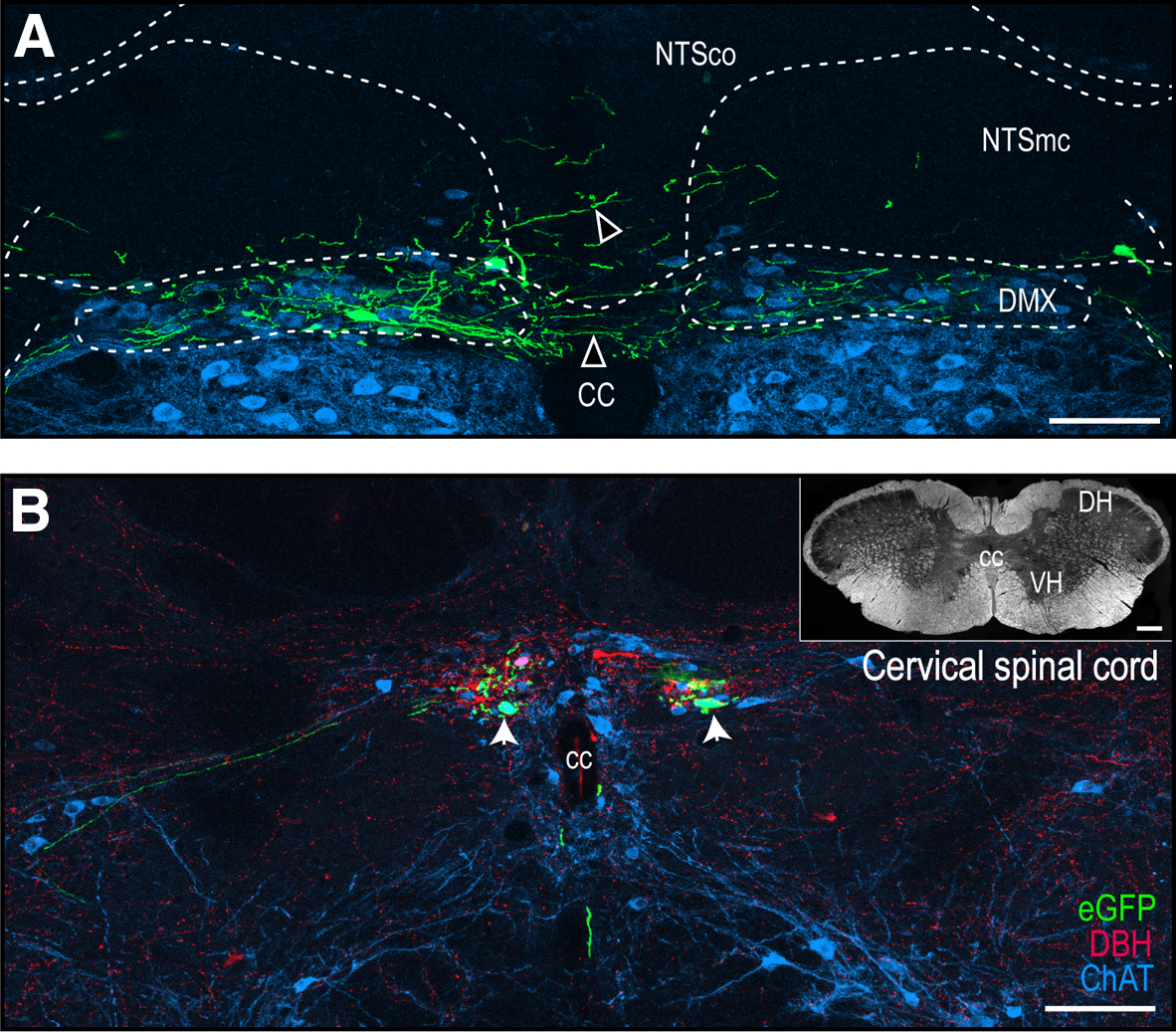
H129-infected neurons in the DVC and cervical spinal cord. Confocal maximum projections of a representative section of the DVC corresponding to Swanson rat brain AL 70 (Swanson, 2018; ***A***) and of eGFP-labeled structures at a representative cervical spinal cord section (***B***). Open arrowheads show eGFP-labeled neurons that cross the midline or extend dorsally from the DMX to the NTS (***A***). White arrowheads indicate double-labeled eGFP + ChAT somata. Green channel, eGFP; red channel, DBH; blue channel, ChAT. Dark-field photomicrograph of the complete cervical spinal cord section is shown in ***B***. Scale bars: 100 μm (***A***, ***B***) and 200 μm (***B***, inset). cc, central canal; DH, dorsal horn; DMX, dorsal motor nucleus of the vagus nerve; NTSco, commissural part of the NTS; NTSmc, caudal subzone of the medial part of the NTS; VH, ventral horn.

Figure 4 shows the locations of H129-infected neurons as well as their co-occurrence with catecholaminergic and/or cholinergic markers in the DVC after injections into the PMV wall. We found that virtually all H129-infected neurons were restricted to the DMX with only occasional infected cell bodies in the part of the NTS immediately dorsal to its border with the DMX (identified using the locations of ChAT-ir neurons). H129-infected neurons were located across the complete rostrocaudal extent of the DMX, including regions in the cervical spinal cord (Fig. 5*B*). They were most abundant in the mid-levels of the DVC at AL 67–70 and accounted for ∼70% of all H129-infected DVC neurons (Fig. 4*G*,*I–K*). The numbers of H129-infected neurons were asymmetric with ∼50% more neurons in the left DMX compared with the right (Fig. 4*L*). Although the numbers of neurons containing eGFP-ir varied greatly between PMV wall-injected animals, neuronal locations in all animals were consistent with those shown in Figure 4. Please note that we present evidence later in Discussion for excluding the delayed retrograde transport of H129 as a contributor to the DMX labeling we describe here.

**Figure 6.**
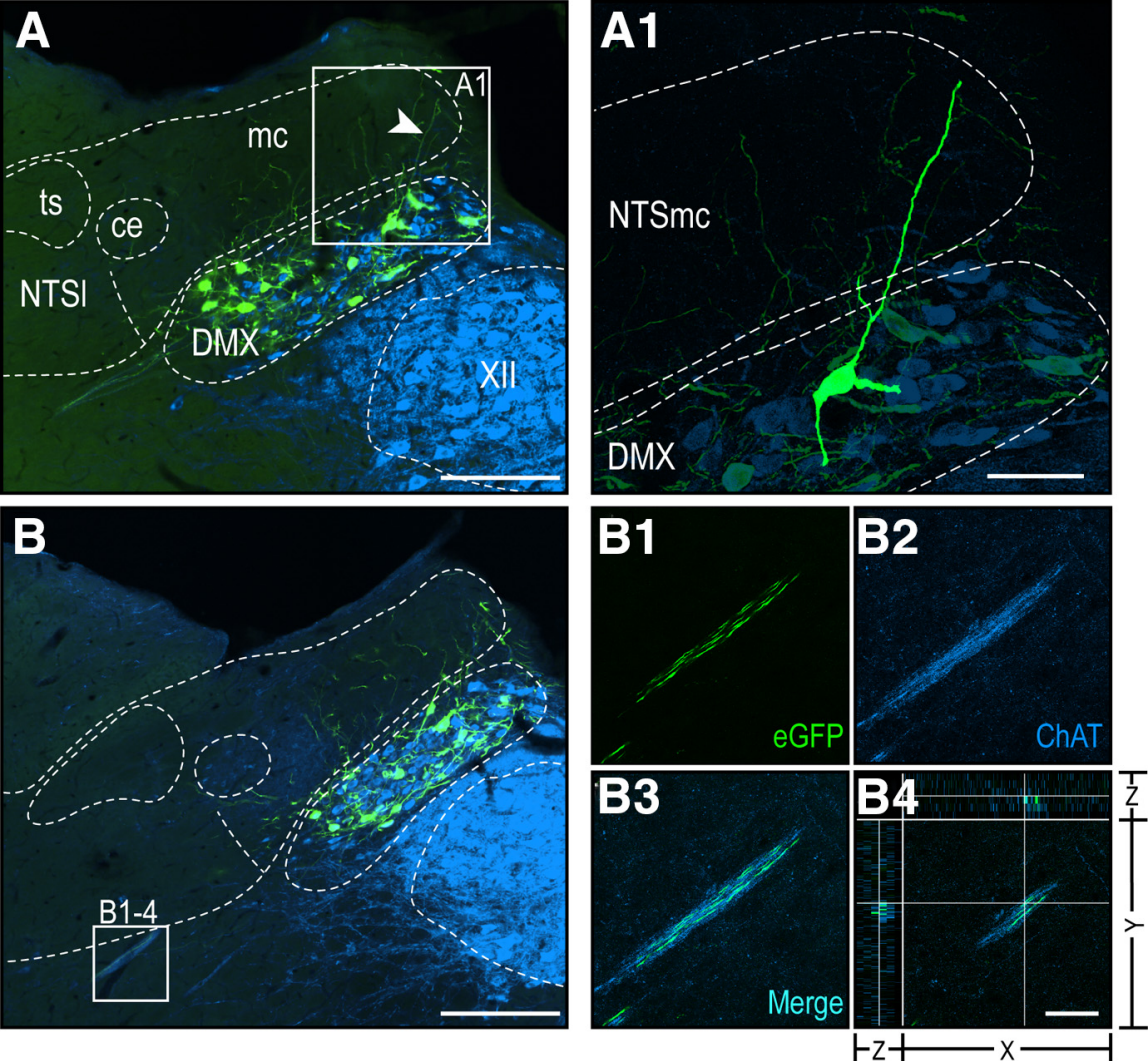
Co-occurrence of H129 with ChAT in DMX neurons of PMV wall-injected rats. Epifluorescence photomicrographs showing dendrites (***A***) or axons (***B***) of the DVC corresponding to Swanson rat brain AL 67 (Swanson, 2018). Confocal maximum projection of the DVC showing a dendrite from a DMX neuron (white arrowhead in ***A***, which is also shown at higher magnification in ***A1***), that extends dorsally to NTSmc territory. Confocal maximum projections of eGFP-labeled (***B1***) and ChAT-labeled (***B2***) axons. Panel ***B3*** shows the merged image where single labeled structures are in green or blue, and co-occurring structures in cyan. Three plane orthogonal view is shown in ***B4*** indicating the co-occurrence of eGFP and ChAT. The arrangement of the *x*-, *y*-, and *z*-axes is shown on panel ***B4***. Green channel, eGFP; blue channel, ChAT. Scale bars: 200 μm (***A***, ***B***), 50 μm (***A1***), 40 μm (***B1–B4***). DMX, dorsal motor nucleus of the vagus nerve; NTSce, central part of the NTS; NTSl, lateral part of the NTS; NTSmc, caudal subzone of the medial part of the NTS; ts, solitary tract; XII, hypoglossal nucleus.

Dendrites of H129-infected neurons in the DMX often extended appreciable distances beyond its border. At levels containing the AP (AL 69–73), these dendrites extended laterally across the midline into the contralateral DMX, or dorsally into the commissural and medial-caudal parts of the NTS toward, and in some cases closely approached the AP (Figs. 4*J–O*, 5*A*, 6*A*,*A1*).

Preganglionic DMX neurons are topographically organized in columns that control different gastrointestinal organs (Fox and Powley, 1985). However, despite H129 infections being distributed along the entire rostrocaudal extent of the DMX (Figs. 4, 5), including its extension into the cervical spinal cord (Dennison et al., 1981), we were unable to discern any notable columnar organization to PMV H129 infections.

### Co-occurrence of H129 with cholinergic and catecholaminergic neurons in the DVC

Consistent with the vast majority of DMX neurons being cholinergic, we found that in the two most extensively labeled cases 93% of all H129-infected neurons in the DMX also contained ChAT (Fig. 4*D*,*H*,*L*,*P1–P4*). However, 6% of H129-infected ChAT neurons also contained DBH. These triple-labeled neurons were in the rostral DMX (AL 63–66) in a region that was closely located to noradrenergic A2 neurons (Fig. 4*C–F*). In more medial and caudal parts of the DMX (AL 67–73), the majority of H129-infected neurons co-occurred with ChAT (96%) but not DBH (Fig. 4*G–P*). We also found eGFP-ir in some ChAT-containing axons that appeared to originate from cholinergic DMX neurons indicating H129 infections in their output axons (Fig. 6*B*,*B1–B4*). We did not find eGFP-ir in any DBH-containing axons.

### Spinal and vagal sensory neurons both contribute to H129 PMV wall infections in the brain

**Figure 7.**
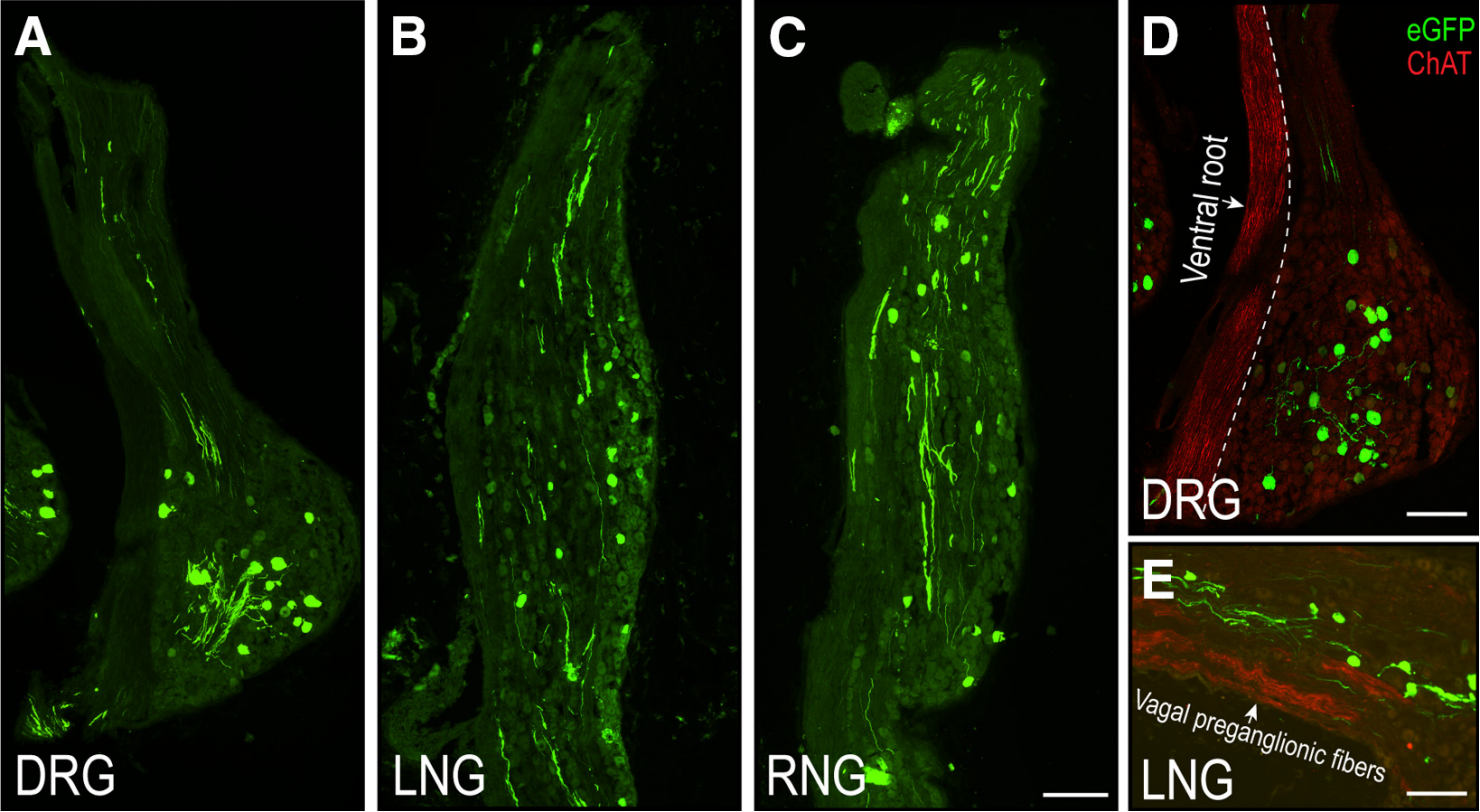
H129-infected sensory neurons in nodose and DRGs of PMV wall-injected rats. Confocal photomicrographs of DRG (***A***), LNG (***B***), and right nodose ganglion (RNG; ***C***) showing sensory neurons labeled with eGFP. Panels ***D***, ***E*** show that eGFP-labeled neurons (green channel) in the DRG (***D***) and LNG (***E***) are in completely separate locations to the cholinergic (ChAT, red channel) motor axons. Scale bars: 200 μm (***A–D***) and 300 μm (***E***).

We found H129-infected neurons and fibers in the thoracic DRGs, and in both nodose ganglia of PMV wall-injected animals (Fig. 7*A–C*) confirming that both spinal and vagal sensory routes were taken by H129 to reach the DVC. ChAT-ir axons seen in the sections containing the DRG and nodose ganglia were confined to the ventral roots of spinal motor nerves that are located adjacent to the DRGs (Fig. 7*D*), and to the vagal motor nerves in the nodose ganglia that run parallel to but are separate from the vagal sensory nerves (Fig. 7*E*). No eGFP-ir was evident in any of these axons.

**Figure 8.**
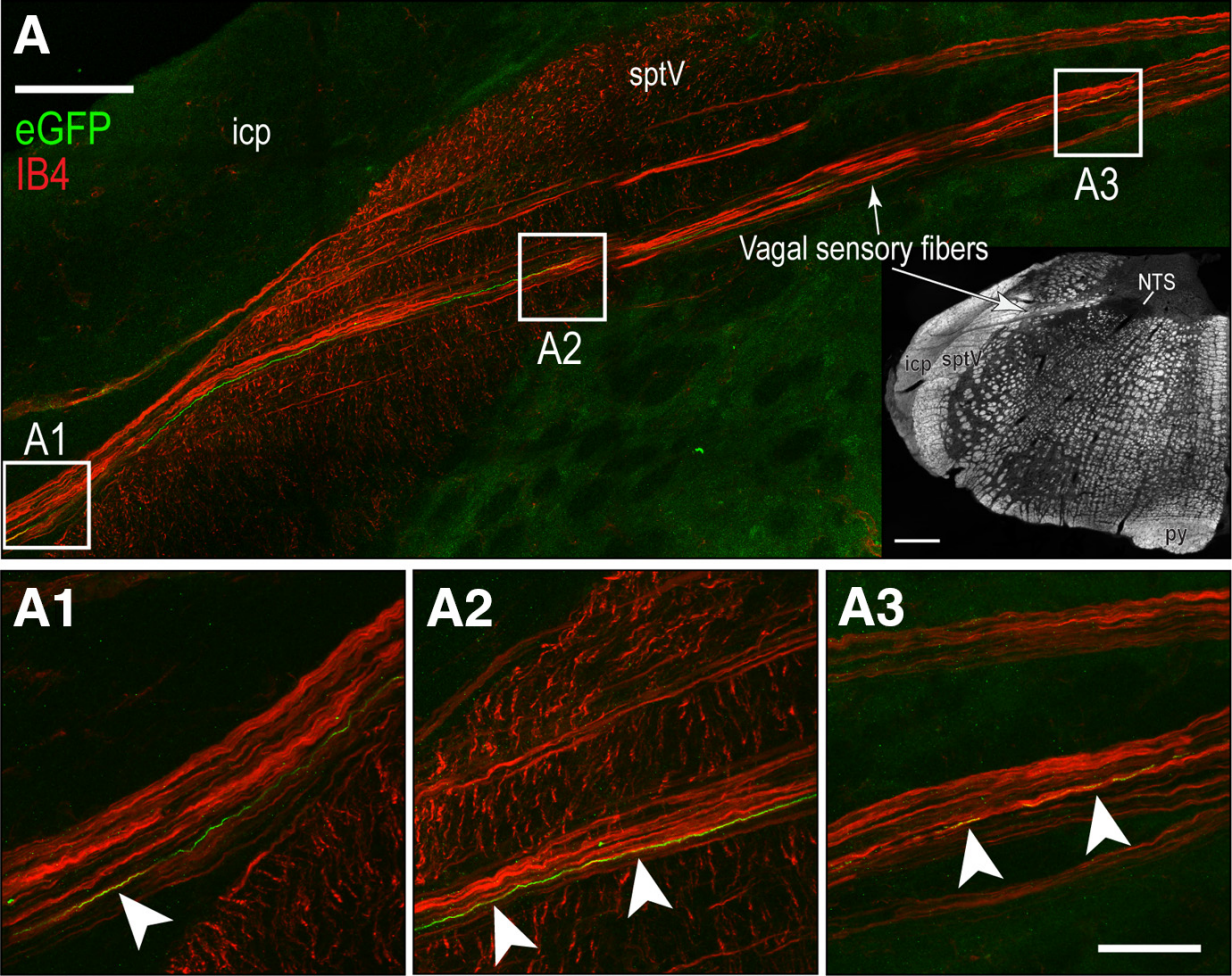
Vagal sensory axons in PMV wall-injected rats. Confocal maximum projection showing H129-infected axons (eGFP, green channel) and the primary vagal sensory neuronal marker IB4 (red channel). The intramedullary path taken by vagal sensory axons to reach their targets in the DVC is clearly evident (white arrows). Low magnification of dark-field photomicrograph (panel ***A***, inset; Swanson rat brain AL 62 (Swanson, 2018) shows the pathway followed by the left sensory vagus nerve. Panels ***A1–A3*** show higher magnification images of the regions indicated by the white boxes in panel ***A***. Co-occurring fibers are seen in yellow and are indicated by the white arrowheads. Scale bars: 100 μm (***A***), 40 μm (***A1–A3***), and 200 μm (***A***, inset). icp, inferior cerebellar peduncle; NTS, nucleus of the solitary tract; sptV, spinal tract of the trigeminal nerve.

H129-infected vagal sensory fibers co-occurred with the primary sensory nerve marker IB4 in the medullary dorsal root entry zone of vagal sensory fibers of PMV wall-injected rats. eGFP-ir fibers of PMV wall-injected animals were evident at the dorsolateral root entry zone of vagus nerve and along the intramedullary path to the DVC co-occurring with the vagal sensory marker IB4 (Fig. 8*A*,*A1–A3*). These H129-infected vagal sensory axons were only seen at the left entry zone of the ventral root.

### Vagal sensory appositions to DMX neurons and their dendrites in the NTS

**Figure 9.**
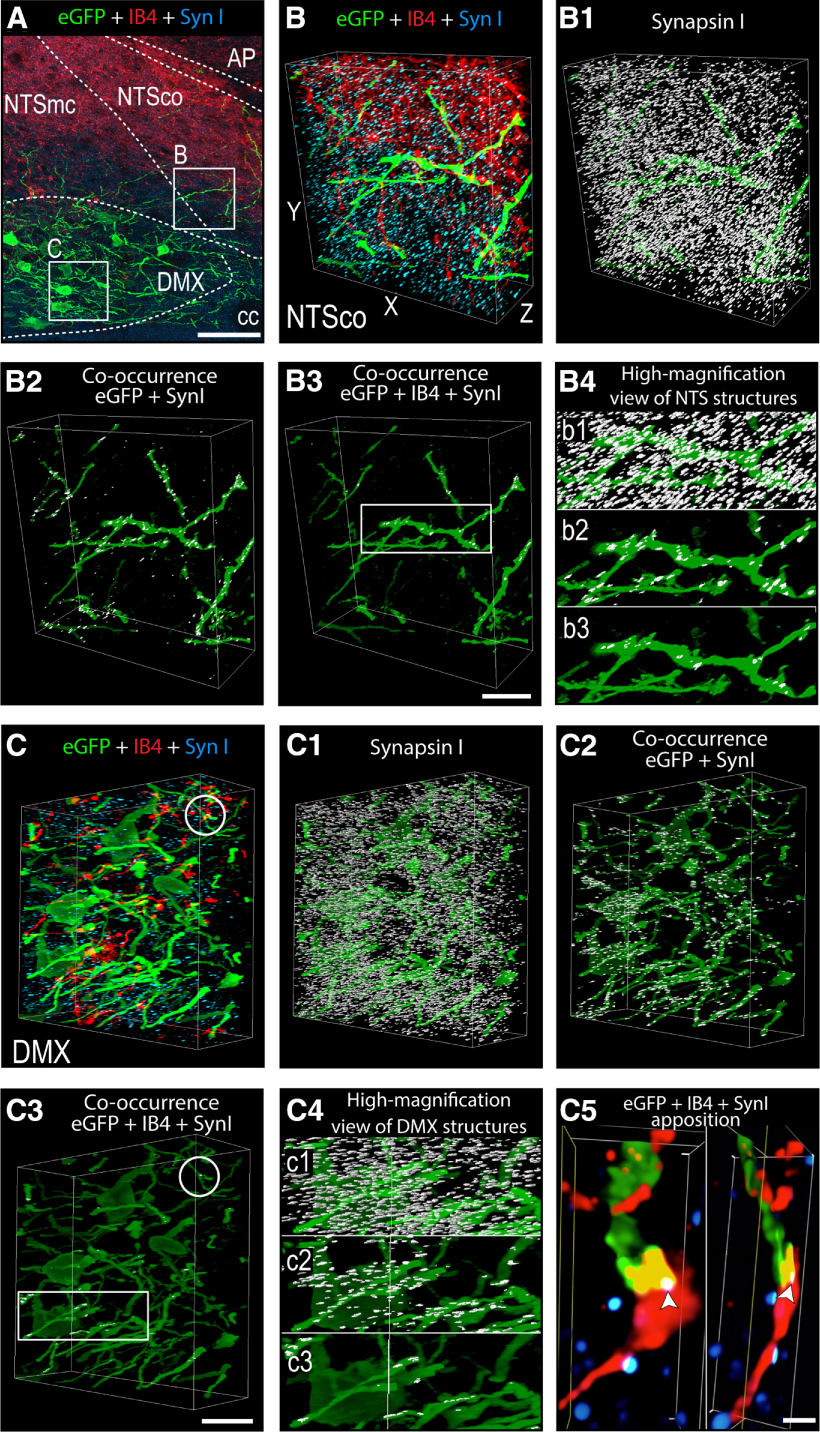
Vagal sensory appositions with H129-infected structures in the DVC of PMV wall-injected animals. Confocal maximum projection (***A***) of eGFP (green channel), IB4 (red channel), and SynI (blue channel) labeling. 3D confocal reconstructions of 123 layers of a 30-μm Z-stack obtained from regions of the NTSco (***B***) and the DMX (***C***) indicated by the white boxes in ***A*** showing eGFP-labeled, IB4-labeled, and SynI-labeled structures (***B***, ***C***). 3D confocal reconstruction of eGFP signal (green) and SynI (white mask; ***B1***, ***C1***), and of eGFP signal and the co-occurrence of double-labeled and triple-labeled structures in the NTSco (***B2***, ***B3***) and the DMX (***C2***, ***C3***). High-magnification view of ***B1–B3*** NTS (***B4***) and ***C1–C3*** DMX (***C4***) structures depicted in the white rectangle in ***B3***, ***C3***. Panel ***C5*** shows a 3D confocal image of a vagal apposition (IB4 + SynI) to a H129-infected structure (eGFP) indicated by the white circles in ***C***, ***C3***, derived from a 98 layer of a 13-μm Z-stack. The location where the three markers co-occur is white and indicated by white arrowheads. The arrangement of the *x*-, *y*-, and *z*-axes is shown on panel ***B***. Scale bars: 20 μm (***A***, ***B3***, ***C3***) and 1 μm (***C5***). AP, area postrema; cc, central canal; DMX, dorsal motor nucleus of the vagus nerve; NTS, nucleus of the solitary tract; NTSco, commissural part of the NTS; NTSmc, caudal subzone of the medial part of the NTS.

Figure 9 shows that eGFP, IB4, and SynI-ir were found in the medial and commissural NTS, and in the DMX. Thus, by using SynI to delineate axon terminals (Fig. 9*B1*,*C1*) we were able to create white masks that identified those structures where SynI co-occurred with eGFP-ir and/or IB4 (Fig. 9*B2–B4*,*C2–C4*). Not only did we find vagal sensory axons from the PMV wall terminating in the medial and commissural NTS but also in the DMX (Fig. 9*A–C*). Additionally, we found more eGFP-ir and SynI appositions with dendrites and cell bodies in both the commissural NTS and the DMX (Fig. 9*B2*,*C2*) than those containing all three labels (eGFP, IB4, and SynI) in the same regions (Fig. 9*B3*,*C3*and *B4*,*C4*). However, the existence of triple-labeled terminal appositions clearly showed vagal sensory neurons from the PMV wall terminate on DMX dendrites in the commissural NTS and DMX, as well as on DMX somata.

## Discussion

Our principal finding is that chemosensory information from the PMV wall is conveyed by vagal and spinal routes to a group of neurons located along the entire rostrocaudal extent of the DMX, but predominantly on its left side. Although we found evidence of H129-infected spinal and particularly vagal (i.e., IB4-containing) terminal appositions in the NTS, the dearth of H129-infected NTS neuronal somata supports a route into the DMX that does not include NTS neurons. Moreover, we found evidence of H129-infected vagal sensory terminal appositions on dendrites and neuronal somata in the DMX itself, as well as on DMX dendrites that extend into the NTS. Before discussing the anatomical and functional aspects of our findings, we first consider the technical constraints of our methods.

### Technical considerations

H129 has been used in different mammals to map neuronal networks that receive sensory information from the nodose ganglia (Han et al., 2018), visual system (Archin and Atherton, 2002), stomach (Rinaman and Schwartz, 2004; Horn et al., 2014), white and brown adipose tissue (Song et al., 2009; Ryu et al., 2017), and airway pathways (McGovern et al., 2012, 2015). These studies used different survival times and reported infections of variable severity and prevalence that are dictated by the different injection sites. Furthermore, Lo and Anderson (2011) reported that, depending on their injection sites, 20% to almost 50% of their H129-injected animals never developed any detectable sign of infection. Although most of our animals developed H129 infections in the DVC, their intensity and abundance, but not their locations, was highly variable among animals, observations that are consistent with previous studies.

Although H129 is transported primarily in an anterograde direction, it does have a delayed and less efficient non-transsynaptic retrograde component when injected peripherally. Importantly, however, this is limited to the primary neurons of the sensory chain (Archin and Atherton, 2002; Rinaman and Schwartz, 2004; McGovern et al., 2012; Wojaczynski et al., 2015). When considering evidence of retrograde transport, we believe that the following sets of results exclude the possibility that the monosynaptic retrograde component of H129 transport accounts for any H129 infections in the DVC.

First, while the HPV wall receives some sympathetic motor innervation, any postganglionic parasympathetic motor innervation is limited to occasional cholinergic endings in the HPV wall after it enters the liver in the porta hepatis (Reilly et al., 1978; Berthoud et al., 1992; Akiyoshi et al., 1998; Schäfer et al., 1998). Most importantly, retrograde transport of H129 does not proceed beyond first-order neurons (i.e., it is only monosynaptic). Because DMX neurons are preganglionic parasympathetic neurons, and therefore do not innervate the final target organ (in this case the PMV wall) they cannot be infected by retrograde transmission. However, we do note that the locations of parasympathetic ganglia within the liver are unknown (Berthoud, 2004) meaning that we cannot know how close these may be to the porta hepatis. All our injections were placed in the HPV wall outside the liver (Fig. 1) to avoid those parts of the porta hepatis that may contain occasional parasympathetic motor fibers. Furthermore, those injections that were the closest to the porta hepatis (Fig. 1, group 1), and therefore the closest to possible vagal motor endings, were the least effective at generating brain infections (Table 1), group 3 animals that were injected in the SMV wall, and so the furthest from the porta hepatis, were the most successful. Second, we used two controls to account for any infection that may have derived from the spread of H129 away from the targeted injection sites: H129 injections into the PMV lumen, and application of H129 onto the PMV surface. Only one PMV surface application generated any labeled neurons in the brain, but this animal had an identical infection pattern to PMV wall-injected animals. Figure 1 shows that abdominal white adipose tissue (WAT) is the most abundant tissue adjacent to the PMV. But given that WAT is not innervated by parasympathetic motor neurons (Bartness et al., 2014; Ryu et al., 2017) inadvertent spread of H129 into adjacent WAT cannot provide a source of retrograde labeling in the DMX. Third, in those animals that had significant infections in the DVC after H129 PMV wall-injections we found abundant eGFP-ir in spinal neuronal somata and fibers in the DRG, and vagal neuronal somata and fibers in nodose ganglia (Fig. 7). Fourth, given that vagal preganglionic but not sensory neurons are cholinergic (Barber et al., 1984; Schäfer et al., 1998) we could not find any co-occurrence of ChAT and eGFP-ir in vagal preganglionic axons that are located adjacent to vagal sensory elements in the nodose ganglia (Fig. 7). Cholinergic spinal motor axons run in the ventral roots that are located adjacent to the DRGs (Bellier and Kimura, 2007). Again, we found no co-occurrence of eGFP-ir and ChAT-ir in these neurons. This is entirely expected given that these axons target the skeletal trunk muscles, which are clearly some distance from the PMV wall. Fifth, the co-occurrence of eGFP-ir in IB4-identified vagal sensory axons in the dorsolateral medulla (Fig. 8). Finally, the co-occurrence of eGFP-ir in SynI-identified terminal appositions in the NTS and DMX and importantly, the co-occurrence of eGFP-ir and SynI with the vagal sensory marker IB4 (Fig. 9). We believe that these findings collectively support a transynaptic anterograde-only direction of H129 transport from sensory endings in the PMV wall to axonal terminals in the NTS and DMX.

### Anatomical aspects

#### General observations

The PMV wall is innervated by vagal and spinal sensory endings (Barja et al., 1983; Barja and Mathison, 1984; Berthoud et al., 1992; Akiyoshi et al., 1998; Fujita et al., 2007; Bohland et al., 2014; Mizuno and Ueno, 2017). But given the very limited information about the central targets of PMV wall sensory inputs, we used H129 infections from a time-series of LNG-injected animals to help us determine differences between the distribution pattern of sensory inputs from the PMV wall into medullary regions and that from a wider population of vagal sensory inputs. We found that unlike PMV wall H129 injections, where all DMX infections occurred with very few or no NTS infections (Fig. 2*A*), LNG H129 injections always infected the NTS (Figs. 2*B*,*C*, 3). Furthermore, DMX infections originating from these LNG H129 injections never occurred without the presence of infected NTS neurons, results that are consistent with those of Han et al. (2018), which also demonstrates consistency between rats and mice. These observations are also consistent with previous reports describing the DVC targets of vagal sensory nerves (Kalia and Mesulam, 1980; Kalia and Sullivan, 1982; Rogers and Hermann, 1983; Neuhuber and Sandoz, 1986; Han et al., 2018; Kim et al., 2020).

#### Vagal sensory nerve projections from the PMV wall to the DVC

The majority of vagal sensory nerves from the PMV wall travel in its common hepatic branch, and from there into the left cervical vagus nerve. However, the celiac branch and thereafter the right cervical vagus apparently does provide a minor contribution (Berthoud and Neuhuber, 2000; Berthoud, 2004). Consistent with this organization we found H129-infected neurons in the LNG of all animals that had significant infections in the DVC (Fig. 7). Furthermore, H129-infected vagal fibers were seen at the left, but not the right, root entry zone into the dorsolateral medulla (Fig. 8), suggesting that the left vagus sensory nerve innervates the PMV wall more prominently than the right. Although we also found infections in the right nodose ganglion (Fig. 7), we cannot comment on whether these originated from the celiac branch of the vagus nerve or whether they traversed from the left to the right vagus via the thoracic communicating branch, which provides crossover access of sensory axons from one vagus nerve to the other (Sauter et al., 1983).

**Figure 10.**
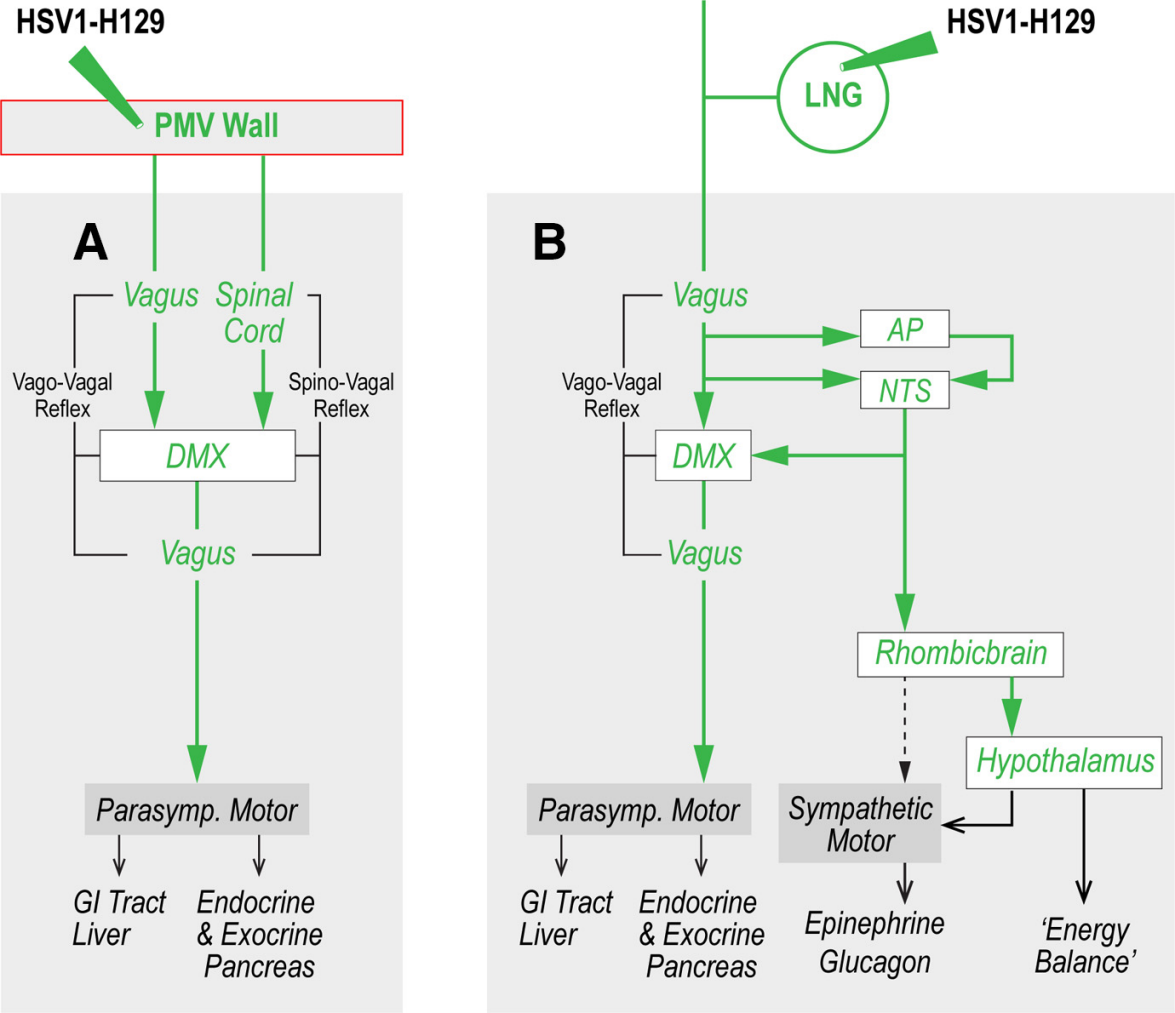
Schematic diagram illustrating the different brain targets of chemosensory information from the wall of the PMV and viscerosensory information conveyed by neurons in the LNG. H129 injections in the PMV wall result in the infection of two neuronal groups in the medulla. These two groups therefore receive chemosensory information from the PMV wall. The first one (***A***) comprises preganglionic parasympathetic neurons located in the DMX that receives both vagal and spinal sensory inputs thereby enabling vago-vagal and spino-vagal parasympathetic reflexes. In turn, these reflexes control gastrointestinal tract, hepatic, and pancreatic function. LNG injections (***B***) show that at least two subsets of vagal sensory inputs may exist: those that terminate in the NTS and AP, consistent with classic sensory systems; and those that derive from PMV wall that terminate exclusively in the DMX. The first group enables ascending projections to integrate vagal sensory information more widely in the brain (also see Krieger et al., 2018), while the second controls parasympathetic vago-vagal reflexes. AP, area postrema; DMX, dorsal motor nucleus of the vagus nerve; HSV1-H129, herpes simplex virus-1 strain H129; LNG, left nodose ganglion; NTS, nucleus of the solitary tract; PMV, hepatic portal and superior mesenteric veins.

Vagal sensory axons terminate in the gelatinous, medial, and commissural parts of the NTS, as well as in the DMX (Kalia and Mesulam, 1980; Kalia and Sullivan, 1982; Rogers and Hermann, 1983; Neuhuber and Sandoz, 1986; Kim et al., 2020). We find the vast majority of eGFP-containing neurons in the DMX co-occurs with ChAT, along with terminal appositions containing eGFP, SynI, and IB4 in the DMX (Fig. 9). These findings are consistent with a direct PMV wall vagal sensory termination onto these preganglionic parasympathetic neurons (Fig. 10*A*). We also find eGFP-containing terminal appositions in the medial and commissural NTS (Fig. 9). However, there are very few H129-containing neuronal somata in the NTS (Figs. 3–5), showing clearly that the main targets of sensory information from the PMV wall are neurons in the DMX not the NTS (Fig. 10*A*). In this way, vagal sensory neurons from the PMV wall must form a subset of those in the LNG that does not terminate on NTS neurons (Figs. 2, 3, 10), thereby indicating a previously underappreciated degree of heterogeneity in the brain targets of vagal sensory neurons.

Direct PMV wall-vagal sensory projections to DMX neurons are most easily explained by the presence of eGFP-containing terminal appositions with DMX somata and their dendrites in the DMX, as well as with dendrites of DMX neurons that extend dorsally into the NTS. Importantly, we find many H129-infected DMX dendrites that extend dorsally into the commissural and medial-caudal subregions of the NTS. Given that the vagal common hepatic branch has sensory nerves that originate in the gastrointestinal tract via its gastroduodenal branch (Berthoud et al., 1992), it seems likely that the gelatinous NTS receives gastric sensory terminals, whereas the medial and commissural NTS is the terminal zone for the majority of PMV wall vagal sensory nerves. Two sets of results are consistent with the notion that the NTS is the location where vagal sensory terminals can appose the dendrites of DMX neurons. First, DMX neurons that innervate the gastrointestinal tract have dendrites that extend into the gelatinous part of the NTS where they receive synaptic input from vagal sensory terminals (Shapiro and Miselis, 1985; Rinaman et al., 1989). Second, we find appositions containing eGFP, SynI, and IB4 in the commissural NTS decorating the dendrites of H129-infected DMX neurons (Fig. 9).

#### Spinal sensory nerve projections from the PMV wall to the DVC

Three sets of results evince that spinal sensory inputs play an important role in conveying PMV wall chemosensory information into the medulla. First, the ability of visceral stimuli to excite DMX neurons by way of direct spinal sensory inputs is blocked by lesioning DRGs (T6–T12; Renehan et al., 1995). Second, those PMV glucosensors important for detecting hypoglycemia have both spinal and vagal origins (Niijima, 1983; Campfield and Smith, 2003; Saberi et al., 2008; Donovan and Watts, 2014). Third, nerve section experiments clearly show that some glucosensing information from the PMV wall reaches the medulla via spinal splanchic sensory nerves rather than the vagus nerve (Fujita and Donovan, 2005; Bohland et al., 2014).

To support these functions those spinal sensory nerves in the splanchnic nerve that terminate in the PMV wall originate in lower thoracic DRGs (T8–T13; Barja et al., 1983; Barja and Mathison, 1984; Fujita and Donovan, 2005; Donovan and Watts, 2014). The significant numbers of H129-infected neurons we see in these same T8–T13 DRGs (Fig. 7) are consistent with these findings. In turn, T8–T13 DRG neurons terminate on lamina I neurons in the dorsal horn of the spinal cord, which then project to the NTS (Menétrey and Basbaum, 1987; Menétrey and De Pommery, 1991). Whether these same dorsal horn neurons project directly into the DMX has yet to be resolved using monosynaptic tracers.

Our results demonstrate that vagal sensory neurons are well positioned to convey chemosensory information from the PMV wall to the DMX. However, the degree to which a spinally-mediated component from the PMV wall is able to do this is less clear. Thus, although we identified H129-infected neurons in the same T8–T13 DRGs that innervate the PMV wall, the lack of an available marker that is equivalent to IB4 for spinally originating sensory inputs, means we are unable to unequivocally identify spinally originating terminal appositions in the NTS and DMX. However, it is possible that at least some of the eGFP-SynI co-occurring terminals we find in the NTS and DMX that lack IB4 (Fig. 9) are of this type (Fig. 10*B*).

### Functional aspects

The PMV is the direct conduit into the liver for nutrients and oxidizable fuels from the small intestine, and for hormones secreted by the gut and pancreas. While liver parenchyma has no known sensory innervation (Berthoud, 2008), the PMV is a unique vascular compartment that contains a wealth of chemosensory information. This includes blood glucose from hypoglycemia to hyperglycemia (Niijima, 1969, 1983, 1989), osmolality (Lechner et al., 2011), natremia (Morita et al., 1990; Morita and Abe, 2011), lipids (Randich et al., 2001; Cox et al., 2004), proteins (Mithieux et al., 2005; Duraffourd et al., 2012), amino acids (Niijima and Meguid, 1995), and the hormones glucagon (Geary, 1990), GLP-1 (Nakabayashi et al., 1996; Balkan and Li, 2000; Vahl et al., 2007), leptin (Shiraishi et al., 1999), and CCK (Horn and Friedman, 2004). Furthermore, the magnitude and dynamics of some of this PMV wall chemosensory information differ from posthepatic locations, for example blood concentrations of glucose (Strubbe and Steffens, 1977), GLP-1 (Deacon et al., 1996; Holst, 2007), and pulsatile insulin secretory patterns (Matveyenko et al., 2008). The ability to convey this type of information from the PMV provides the brain with an additional perspective of the vascular chemosensory components detected either side of the blood-brain barrier.

After it enters the brain, PMV wall chemosensory information influences at least two important functions: food intake, including flavor preference, and glycemia. Both of these functions are influenced by sensory information conveyed vagally and spinally (Novin et al., 1973; Tordoff and Friedman, 1986, 1988; Geary, 1990; Del Prete and Scharrer, 1994; Campfield and Smith, 2003; Fujita and Donovan, 2005; Bohland et al., 2014; Donovan and Watts, 2014; Goswami et al., 2018; Zhang et al., 2018). With this in mind, the robust H129 infections in the DMX together with the virtual absence of H129 in NTS neurons provide structural support for considering PMV wall chemosensory information as a significant and direct controller of vago-vagal reflexes and possibly spino-vagal reflexes (Fig. 10*A*). Indeed, the way vagal sensory inputs from the PMV wall to the DMX appear to be organized means that controlling this reflex may be the sole function of vagally conveyed PMV wall chemosensory information, which is directed at least in part toward controlling food intake.

The parasympathetic motor component of the PMV/vago-vagal reflex, and possibly a PMV/spino-vagal reflex has the capacity to influence a wide range of functions associated with energy balance, including food intake and glycemia. These include the gastrointestinal tract from the lower esophagus to the transverse colon (Berthoud et al., 1991) where postganglionic enteric neurons (Schemann and Grundy, 1992) can, for example, inhibit or excite gastric smooth muscles to exert fine control of gastric tone and motility (Abrahamsson and Jansson, 1973; Rossiter et al., 1990). Parasympathetic motor control also influences pancreatic exocrine (Love et al., 2007) and endocrine function (Buijs et al., 2001; Taborsky and Mundinger, 2012), particularly insulin secretion (Ahrén and Taborsky, 1986; Nagase et al., 1993), a mechanism that may contribute to the hypoglycemic response to HPV hyperglycemia (Burcelin et al., 2000). Interestingly, increases in HPV glucose and GLP-1 increase pancreatic insulin release via a vago-vagal reflex, and they appear to do so synergistically (Nakabayashi et al., 1996; Balkan and Li, 2000; Burcelin et al., 2001). The liver also has a parasympathetic motor innervation (Pocai et al., 2005a,b; Lam et al., 2007).

### Summary and conclusions

Our results show that the vast majority of medullary neurons that are the first to receive chemosensory information from the PMV wall are preganglionic parasympathetic neurons in the DMX. The negligible number of NTS neurons that are infected after H129 PMV wall-injections means that vagally conveyed information terminates more or less exclusively on DMX neurons thereby enabling a PMV/vago-vagal/parasympathetic reflex (Fig. 10). This information flow may be augmented by PMV wall sensory inputs that reach the DMX from the spinal cord, meaning that both vagal and spinal routes provide direct inputs to the DMX. Further studies are needed to clarify the details of these inputs, particularly those mediated by projections through the spinal cord.

## Synthesis

Reviewing Editor: Douglas Bayliss, University of Virginia School of Medicine Decisions are customarily a result of the Reviewing Editor and the peer reviewers coming together and discussing their recommendations until a consensus is reached. When revisions are invited, a fact-based synthesis statement explaining their decision and outlining what is needed to prepare a revision will be listed below. The following reviewer(s) agreed to reveal their identity: John Campbell, Ruth Stornetta.

This manuscript was re-reviewed and both reviewers were generally pleased with the revisions. However, it was also their judgment that the evidence that the PMV-to-DMV sensory input occurs via a spinal route remains incomplete. The rationale for this concern, which was universally supported in consultation, is presented in comments from Reviewer 2. In short, more evidence would be required to substantiate this claim (e.g., showing projections through the dorsal horn and the spinal cord). In light of these concerns, the recommendation was to soften the claims regarding this spinal pathway.

The full comments are provided below.

Reviewer #1

Advances the Field

The neural circuitry which relays chemosensation at the hepatic portal vein to physiological effectors is not well understood. This manuscript advances the field by identifying a neural circuit connecting portal vein sensory neurons with vagal motor neurons that control digestive tract function. Of special interest is the finding that portal vein sensory input is transmitted to vagal motor neurons directly, rather than through the NTS, which goes against the “textbook” understanding of how viscerosensory input is integrated. This reveals a potentially under-appreciated role for direct or non-vagal sensory inputs to vagal motor reflexes.

Comments

The revision and rebuttal letter have addressed each of my questions and concerns. I thank the authors for their detailed response.

Reviewer #2

Advances the Field

The manuscript illustrates an important afferent pathway for conveying information on nutrient load from gut to brain via a vagal sensory pathway.

Comments

I am still not convinced that the authors have shown a spinal route for the sensory information coming from the PMV to the DMX. Although they do show DRG infected neurons, are there neurons infected in the spinal cord dorsal horn? Are there eGFP fibers running through the spinal cord? This evidence would be necessary if there is a relay through the spinal cord to the DMV. Could the authors identify which spinal level DRGs were infected that would be consistent with what is known about the PMV sensory spinal innervation (T8-T13)?

The authors evidence for the spinal route based on other literature is also not convincing. The authors claim that the Saberi et al. 2008 study shows a spinal route for glucose sensing. In this 2008 paper, portal vein and mesenteric portal vein sensory ending ablation by capsaicin results in a blunting of the counter-regulatory response to hypoglycemia but only when the hypoglycemia is induced slowly rather than rapidly. I do not understand how this finding shows a spinal vs. vagal route for glucose sensing as capsaicin destroys both vagal and sensory afferents (e.g. Browning et al. 2013).

Is there any evidence from the literature of a direct projection from spinal dorsal horn to the DMV?

Perhaps the authors could soften their conclusion about the spinal route. They do present evidence in the DRG infection but lacking any further data, this pathway remains a bit uncertain.
